# Performance of Nano-Silica Modified Self-Compacting Glass Mortar at Normal and Elevated Temperatures

**DOI:** 10.3390/ma12030437

**Published:** 2019-01-31

**Authors:** Sakthieswaran Natarajan, Muthuraman Udayabanu, Suresh Ponnan, Sophia Murugan

**Affiliations:** 1 Department of Civil Engineering, Anna University Regional Campus–Tirunelveli, Tirunelveli 627007, Tamilnadu, India; sophiavarshini1992@gmail.com; 2 Department of Electrical and Electronics Engineering, Francis Xavier Engineering College, Tirunelveli 627007, Tamilnadu, India; umraman@gmail.com; 3 Department of Electronics and Communication Engineering, PSN College of Engineering and Technology, Tirunelveli 627152, Tamilnadu, India; suresh3982@gmail.com

**Keywords:** Self compacting mortar, sand replacement, waste glass powder nanosilica, elevated temperature

## Abstract

This research aims to combine the effects of nanosilica and glass powder on the properties of self-compacting mortar at normal and at higher temperatures. The fine aggregate was replaced by waste glass powder at various percentage levels of 10%, 20%, 30%, 40% and 50%. The mechanical properties of self-compacting glass mortar (SGCM) were studied at elevated temperatures of 200, 400, 600 and 800 °C. Furthermore the effect of sudden and gradual cooling technique on the residual strength of glass mortar was also investigated In order to enhance the behavior of SCGM the nanosilica of 3% by weight of cement was added. From the results it was obtained that the glass powder replacement effectively contributed towards the thermal performance while the addition of nanosilica enhanced the mechanical performance. The enhanced physical properties were obtained mainly at the glass transition temperature thus showing the active participation of glass powders during high temperatures. Moreover the gradually cooled specimens exhibited improved strength characteristics than the suddenly cooled specimens.

## 1. Introduction

Today’s world is moving towards the phase of sustainable construction. The attempts to utilize the waste materials in construction are being extensively carried out not only from the economic point of view but also from the social and ecological view [1]. Waste glass is being generated in millions of tons every year thereby causing a serious environmental threat due to their bio-degradable nature [2]. The chemical composition of the glass powder offers several advantages by improving the properties of the concrete when used as a supplementary cementitious material [3]. Waste glass powder is not an unconventional material to concrete construction. Several studies have also shown that the partial replacement of fine aggregate by glass powder proves to be an economical and feasible solution for the production of concrete and mortars due to the high environmental impacts associated the aggregate extraction [4,5,6]. Glass powders as replacements for binder has also revealed that glass powders can effectively function as a pozzolanic material and can be used in combination with nanomaterials to provide enhanced properties [7]. However the strength of the mortar and concrete was found to decrease due to the incorporation of glass powder [8,9]. Studies also showed improved workability due to glass powder substitution [10,11,12] whereas the workability was also found to decrease with increasing glass powder proportion in the concrete [13]. The improvement in the workability was attributed due to the surface glassy texture and the hydrophobic tendency of the glass powders whereas the reduction in the workability may be due to the angular surface and high specific surface area of the glass powders. 

Though concrete structures are highly resistant to temperatures they suffer from a serious threat due to fire [14,15]. The concrete structures when exposed to fire suffer from severe degradation in its strength parameters [16] and it undergoes explosive spalling and disruption causing severe damage to life and property [17]. Hence the study of physical transformations in the concrete after being exposed to higher temperatures becomes important. Self-compacting concrete usage has increased multifold in the past few decades due to its higher strength variations than conventional concrete [18]. Recent decades focused on the temperature studies on self-compacting concrete [19,20,21,22] and mortar due to the significance of mortar properties of self-compacting concrete since it contains less aggregate [23,24].

Nano-technology is also emerging as a popular field in the construction industry [25] and has been found to increase the strength of concrete [26] and cement mortars [27,28]. Nano materials have created a profound impact on the concrete production due to the novel physical effects caused on the properties of concrete [29]. The supplementary cementitious materials have been found to increase the fire resistant property of concrete [30] and showed significant effects when used in combination with nanomaterials [31,32]. The nanomaterials are a significant hydrating agent and an active by filling the thereby forming compact microstructure [33]. The inclusion of nanomaterials can reduce the porosity of concrete which is a crucial parameter that affects strength of concrete by keeping the moisture intact and thus maintaining the concrete structure and integrity after exposure to high temperatures [34]. Nano-silica has been found to be an effective replacement of cement products and have also exhibited positive results [35]. These strength variations due to nanomaterials are mainly imparted due to the filler effect of these nanoparticles and they also increase the rate of hydration [26]. Even the micro scale additions of nanosilica have found to increase the mechanical and physiological characteristics of cement mortar and concrete as obtained from previous literatures [27].

Numerous research works have already been done by the replacements of waste glass powder and also as partial substitutes for fine aggregates in normal and self-compacting concrete at normal [1,2,3,4,5,6,7,8,9] and higher temperatures [10,11]. This research work mainly aims at utilization of waste glass powder in self compacting mortar with the combined effect of Nano-silica as partial replacement for cement. Despite several studies on glass powder concrete no study has been attempted to characterize the temperature effect on glass powder self-compacting glass mortar. The effect of adding nanomaterials on the self-compacting glass mortars have also not been studied so far.

The effect of elevated temperatures by using recycled glass powder on the self-compacting concrete was already done by Poon et.al [36]. This research work was also conducted similarly but the mortar properties were discussed briefly as well as the effect of nanosilica in modifying the properties of self-compacting glass mortar was investigated. Furthermore the types of cooling technique on the mechanical strength of the SCGM were also examined.

## 2. Materials and Proportioning

### 2.1. Cement

Ordinary Portland cement of grade 43 conforming to BS EN197-1 [37] is used. The brand name is Coromandel Cements. Tests were carried out on various physical properties of cement and the results are shown in Table 1.

### 2.2. Glass Powder

**Figure 1:**
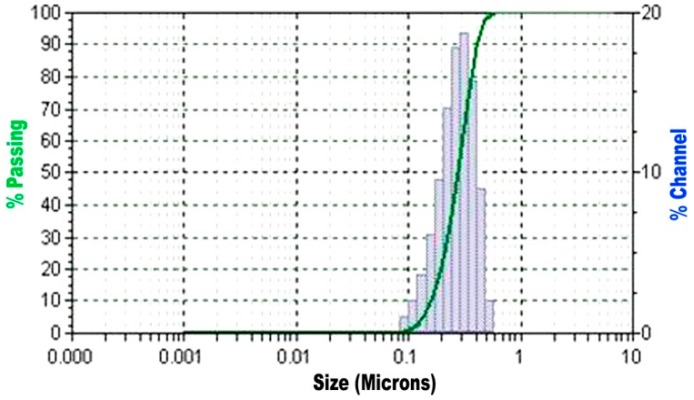
Particle size distribution results of glass powder.

The fine glass powder was used a partial replacement for fine aggregate and the obtained particle size distribution is shown in Figure 1. The chemical composition of the glass powder obtained by ED-XRF analysis is shown in Table 2.

### 2.3. Nano Silica

Nano silica was added 3% by weight of the cement for all the self-compacting mortar mixes. This range was chosen as established in previous works that at this level the properties of cement was very much enhanced [26].

### 2.4. Fine Aggregate

**Figure 2:**
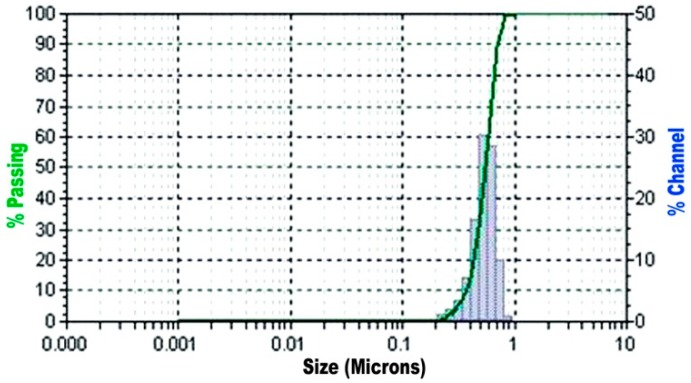
Particle size distribution results of fine aggregate.

Natural river sand was used as fine aggregate confirming to EN 12620-2002 [38]. The results obtained from sieve analysis indicate that the sand conforms to Zone III and the particle size distribution graph of the fine aggregates used is shown in Figure 2. The fineness modulus of sand was 2.49. The specific gravity was 2.58.

### 2.5. Super Plasticizer

Super plasticizer which is a naphthalene based under the trade name of Enfiiq was used to achieve required workability. The dosage of super plasticizer was constant at ~1.5% of weight of cement throughout the mix.

### 2.6. Mix Proportion

The self-compacting mortar was prepared by CEM method [39] as followed in several other previous works [40,41]. The water to cement ratio was maintained constant as 0.4. The superplasticizer dosage was also kept constant to determine the effect of glass powder on the workability of each mix. The proportion of the self-compacting mortar is shown in Table 3.

The self-compacting glass mortar was then prepared by substituting the sand by glass powder at various dosages (10, 20, 30, 40 and 50%) by the weight of fine aggregate. Nano silica was added 3% by weight of the cement in the glass powder replaced mixes.

## 3. Methodology

### 3.1. Tests for SCGM in Fresh State

**Figure 3:**
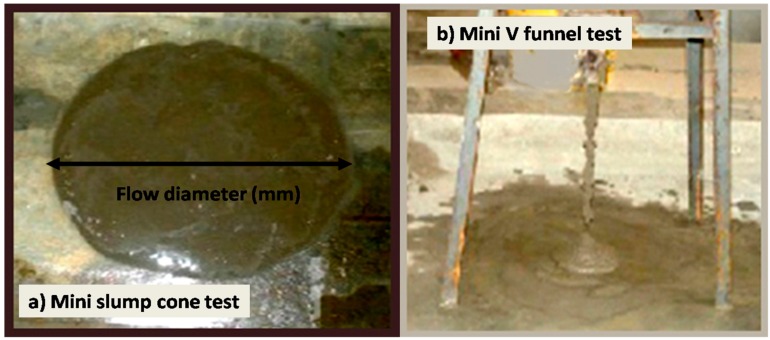
Fresh state behavior of the SCGM. (**a**) Mini slump cone test; (**b**) Mini V funnel test.

The fluidity of the SCGM was evaluated using the mini-cone test and the V funnel flow time test immediately within 5 min mixing of the mortar as shown in Figure 3.

### 3.2. Casting and Curing

The self-compacting mortars after conducting the fresh state tests were then cast into cubic specimens of size 50 × 50 ×50 mm^3^ and for compression testing and prisms of size 160 × 40 × 40 mm^3^ for flexural testing procedure. These samples were left in the mold for 24 hours after which they were demolded and placed under water for curing up to 28 days. After 28 days the specimens were then tested for compression and flexure to determine the mechanical strength of the mortars at normal temperatures.

### 3.3. Heating of the Test Specimens

**Figure 4:**
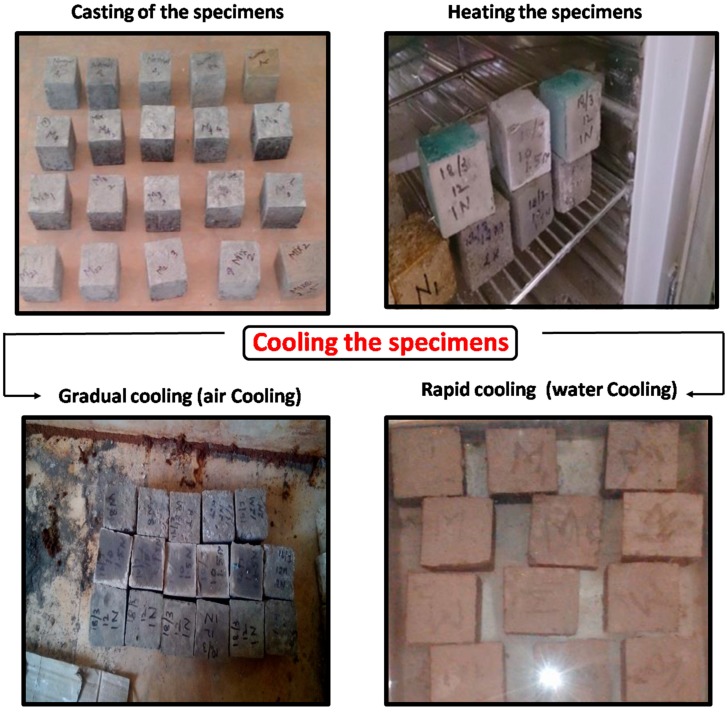
Self-compacting glass mortar specimens.

**Figure 5:**
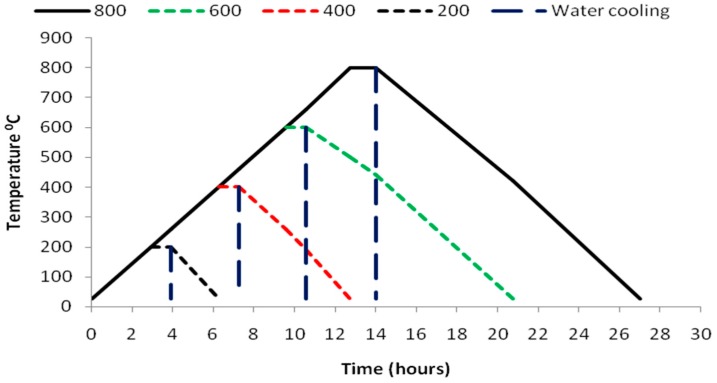
Heating and cooling regimes for Air and water cooling.

The specimens with and without the glass powder replacement were then heated in a muffle furnacecoupled with a digital controller which can attain a maximum temperature of 1000 °C atuniform heating rate of 20 °C per minute. The heating of the specimens were done at a range of 200 °C to 800 °C in increments of 200 °C. The inbuilt thermostat in the muffle furnace automatically regulates the temperature and rate of heating of the specimens. After the oven has reached the target temperature the specimens were left to remain in the oven for about 1 hour and so that the specimens may undergo stabilized heating throughout their cross section The heated samples were then subjected to two types of cooling regimes in which a part of the specimens were cooled in air and the other part were suddenly cooled by immersing in water. For each test three specimens were casted and the results were reported as the average of the three specimens. The produced self-compacting glass mortar specimens are shown in Figure 4 and the heating and cooling regimes for air and water cooling adopted in the present study are shown in Figure 5.

### 3.4. Bulk Density

The bulk densities of self-compacting mortar with and without the glass powder replacements were determined after 28 days water curing of the samples. The variation of the bulk density was determined for all mortars with and without the partial replacement of sand by glass powder after subjected to various elevated temperatures of 200 °C, 400 °C, 600 °C and 800 °C.

### 3.5. Porosity

The porosity was also determined knowing the saturated mass of the samples and mass of cubic samples which were oven dried. The dried mass was obtained after drying saturated samples in an oven at 60 °C until the specimens achieved constant weight. The apparent volumes of the samples were determined using pycnometric method.

(1) $$\mathrm{Porosity}\;\left(\%\right)=\;\frac{\left(\mathrm{Void}\;\mathrm{volume}\;\mathrm{occupied}\;\mathrm{by}\;\mathrm{water}\right)}{\left(\mathrm{Apparent}\;\mathrm{volume}\;\mathrm{of}\;\mathrm{the}\;\mathrm{specimen}\right)}\;\times 100$$

### 3.6. Cube Compression Test

The cube compression tests were conducted as per EN 1015 -11: 1999 [42] in which the water cured samples after 7, 14 and 28 days were tested 400 ton capacity compression testing machine. The compressive strength tests were also done on the specimens after subjecting to the elevated temperatures. The water cooled and the air cooled specimens were tested after respective cooling periods. The load was applied uniformly until the specimens were failed to sustain further loading.

The compressive strength was calculated from the relation

(2) $$\mathrm{Compressive}\;\mathrm{strength}\;\left(\frac{N}{{\mathrm{mm}}^{2}}\right)=\;\frac{\left(\mathrm{Maximum}\;\mathrm{load}\;\mathrm{at}\;\mathrm{failure}\right)}{\left(\mathrm{Cross}\;\mathrm{sectional}\;\mathrm{area}\right)}$$

### 3.7. Flexural Strength

The flexural strengths were done on the prisms of size 160 × 40 × 40 mm^3^ according to ASTM standards C 348-14 [43]. The flexural strengths were done after 28 days of curing for both the glass replaced and unmodified self-compacting mortar. The effect of elevated temperatures on the flexural strength were also done on the specimens after subjected to various heating regimes and cooling methods like rapid cooling by water and gradual cooling by air until the room temperature was reached.

### 3.8. Mass Loss

The mass losses of the specimens after exposure to various temperatures were measure accurately using a weighing balance. The mass loss was recorded as a percentage of initial mass of the specimen before exposure to temperature. The porosity studies indicate that the porosity values decreased for the specimen with the glass powder replacement. However the porosity values were increasing for the specimens prepared with glass powder with smaller proportions. This may be due to the fact that the lesser cement glass powder caused a perfect bending of the cement and glass powders whereas the mortars with the greater glass powder proportion caused a negative influence on the porosity values as shown in Figure 5. Moreover the porosity studies were done only to evaluate the pores in between the cement and glass powders and hence is only a measure of open porosity.

## 4. Results and Discussion

### 4.1. Fresh State Properties

**Figure 6:**
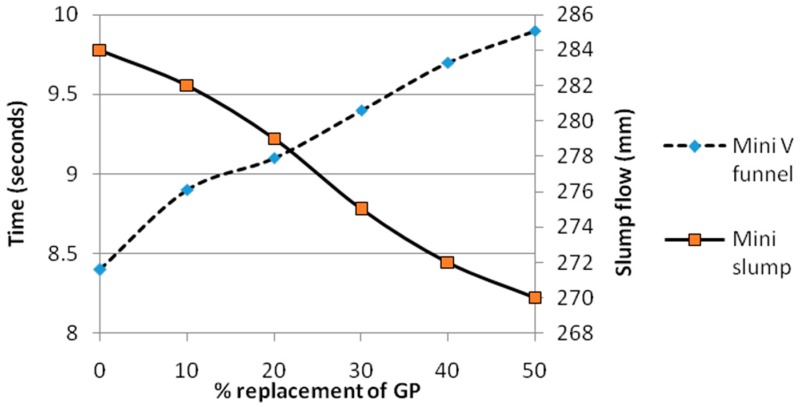
Fresh state results of self-compacting glass mortar.

Figure 6 shows the fresh state properties of self-compacting glass mortar modified using nanosilica plotted as a function of replacement levels of glass powder. The observed results were simply to confirm the workability parameters with reference to the unmodified mix. The self-compatibility of the mixes increased with increase in the glass powder content. However the observed values showed minor decrease in the flow values due to the incorporation of the nanosilica particles in the glass powder mixes. It has been previously established in other research works that addition of nanosilica causes decrease in the spread and flowability of self-compacting mortar mixes [26]. This can be justified by the fact that nanosilica demands more water and thereby reduces the free water availability causing dense structure [32]. 

However the decrease in flowability caused by the addition of nanosilica was nullified by the increasing glass powder content due to the weak inter molecular cohesive force between finer glass particles and due to their smooth surface texture [5]. Furthermore the fresh state results as examined through visual observations as shown in Figure clearly indicated that the segregation and bleeding in the mixes at higher levels of glass powder replacement was very much reduced due to the effect of nanosilica. These results proved the beneficial effects of nanosilica in arresting segregation and bleeding that were the major drawbacks observed at 40 and 50% replacement of glass powder as reported by Ali et al. [4].

### 4.2. Bulk Density

**Figure 7:**
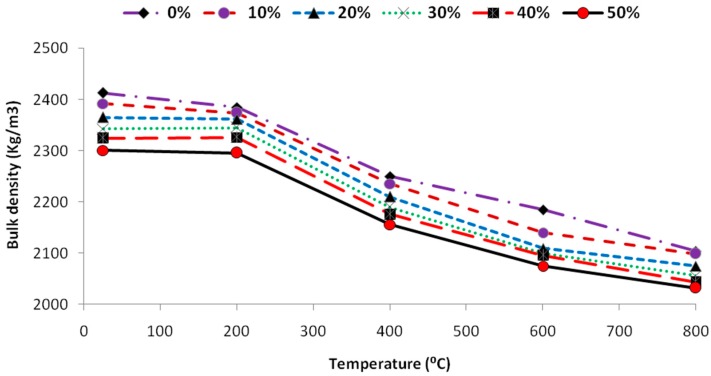
Density variations of SCGM at various temperatures.

The density of the self-compacting glass mortars decreased with increased glass content as shown in Figure 7. This relative decrease in the density was due to the lower density of the glass powder than the fine aggregate (sand). Furthermore the increasing temperature caused lower density of the samples and the density variation was pronounced at the temperature above 400 °C. These density variations at higher temperatures above 600 °C were almost similar for all the samples irrespective of the content of the glass powder.

### 4.3. Porosity

**Figure 8:**
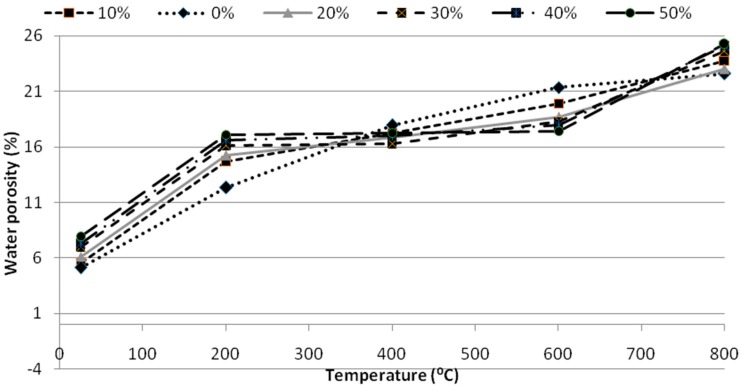
Porosity percentages of the SCGM at various temperatures.

The porosity studies indicate that the porosity values decreased for the specimen with the glass powder replacement. However the porosity values were increasing for the specimens prepared with glass powder with smaller proportions. This may be due to the fact that the lesser cement glass powder caused a perfect binding of the cement and glass powders whereas the mortars with the greater glass powder proportion caused a negative influence on the porosity values as shown in Figure 8. Moreover the porosity studies were done only to evaluate the pores in between the cement and glass powders and hence is only a measure of open porosity. The reduction in the porosity may be due to the increased fineness of the glass powders that filled the voids created due to the loss of moisture from the concrete at increasing temperatures which is the cause of occurrence of pores in concrete after exposure to higher temperatures. This may be due to the capacity of the fine particles of glass. Furthermore it can be explained by the synergetic effect of the nanosilica and glass powder in reducing the porosity. The filler effect of nanosilica contributed to the porosity reduction especially at high temperatures.

### 4.4. Compressive and Flexural Strength

**Figure 9:**
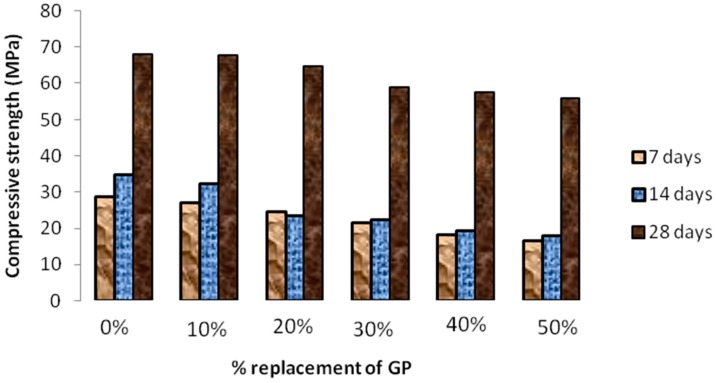
Compressive strength values of SCGM at various periods.

Compressive strength results of all mortar samples that were prepared by the partial replacement of fine aggregates by the glass powder are shown in Figure 9. These results were in accordance with the previously published literature [16]. However the strength values obtained were higher than the other research works for the same percentage of glass powder content. This strength increase proved the filler effect of nanosilica that filled the voids created between the glass powder and the fine aggregate (sand). Thus the increase in the compressive strength values was obtained at each mix proportion than those values obtained by the other research works [12].

**Figure 10:**
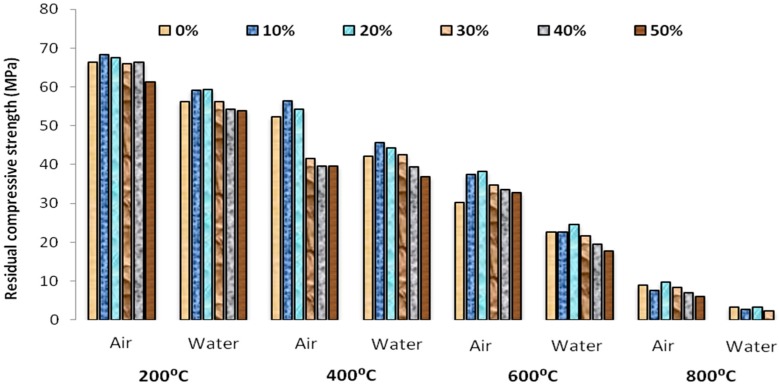
Compressive strength of SCGM at high temperature subjected to air and water cooling.

The residual compressive strengths of the mortar specimen after subjected to high temperatures were shown in Figure 10. The effect of cooling regimes on the glass mortar was also shown. The reference mortars without the replacement of glass powder were also presented so as to compare the results. The replaced samples show a comparatively lower strength when compared to the reference samples but these strength variations are however negotiable. The 7 days strength showed a lesser value than the 28 days for each value of the replaced samples. However, the substitution of fine aggregate by glass powder showed a linear decrease in the compressive strengths of Self compacting mortar. This clearly proves that the compression strength reduced with increase in the proportion ratio of glass powder which is in accordance with the previous works [19].

The compressive strength values at higher temperature are also shown in Figure 10. From the obtained values it can be predicted that the glass powder substitution caused a positive effect on the temperature resistance of self-compacting mortar. The strength variation with the increase in temperature was higher for the glass powder replaced mortar than the reference mortar. The reduced strength of the glass powder replaced mortar subjected to sudden cooling may be due to the thermal shock caused in the glass powder that had undergone a transition from the glassy state to the rubber like structure. Moreover the cement paste shrinks at high temperatures whereas the aggregate phase expanded due to the temperature increase which caused the strength decrement. Though the compressive strength value reached the lowest value at the attainment of 800 ºC the performance of the glass powder self-compacting mortar exhibited better values than the traditional self-compacting mortars. This may be due to the chemical composition of the glass powders which is composed of amorphous silicon dioxide which is highly thermally stable.

The residual compressive strength of the self-compacting glass mortar specimens after heated to 200 °C were higher than the reference specimens for all concentration of glass powder. This observed increase in strength variation at this temperature was due to the addition of nanosilica which caused an increase in the compressive strength due to the hydration of unhydrated particles in the cement and due to the improvised mechanical properties of calcium silicate hydrate gel. These increased compressive strength results obtained at 25–200 °C were contrary to the results obtained by Poon et al. [19]. This showed that nanosilica added self-compacting glass mortar exhibits higher thermal stability.

**Figure 11:**
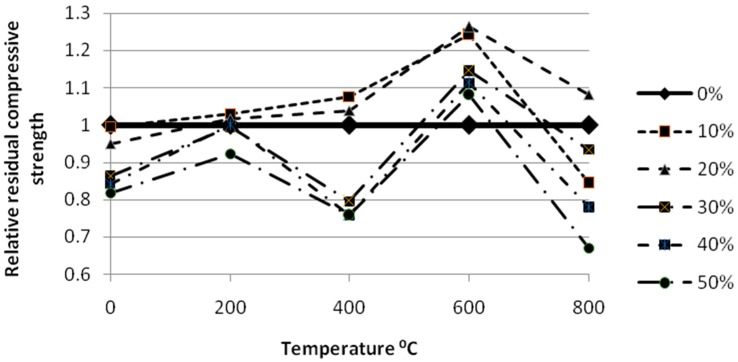
Relative residual compressive strength of gradually cooled SCGM at high temperature.

The air cooled specimens exhibited a little strength variation than the water cooled specimens and this may be due to the gradual cooling of the specimens rather than the sudden cooling which may have caused this strength variation. The relative residual compressive strength of gradually cooled self-compactingglass mortar at high temperature was shown in Figure 11.

**Figure 12:**
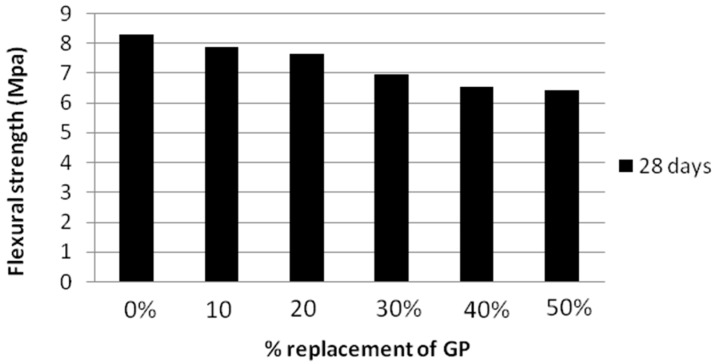
Flexural strength values of SCGM at various percentages of glass powder.

The variation of flexural strength with various amounts of glass powder was shown in Figure 12. From the results it was proved that the flexural strength decreased with increase in percentage of glass powder in self compacting mortar mixes. According to the results, the decrease in the flexural strength compared with the reference mix was 5.1%, 8%, 16%, 21%, 22.5% for 10%, 20%, 30%, 40% and 50% replacement levels respectively. These results were better than the results obtained by Ali et al. [5], which clearly explained the favorable effects of nanosilica in neutralizing the negative effect of glass powder on the flexural strength of glass mortar.

**Figure 13:**
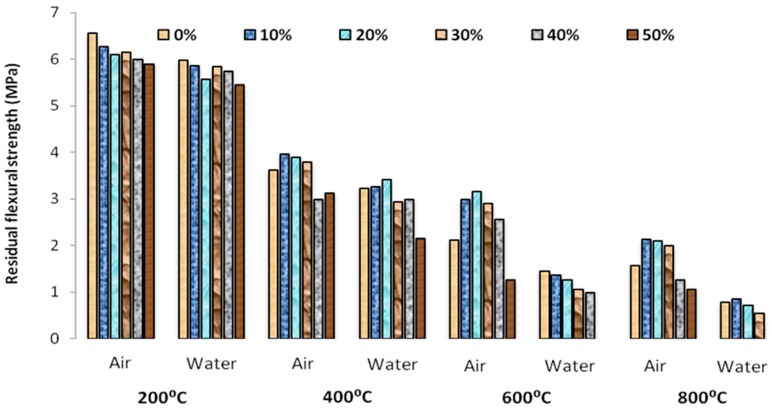
Residual flexural strength of SCGM at high temperature subjected to air and water cooling.

The residual flexural strength of the self-compacting mortar with various percentage of glass powder replacement after heating to four stages 200 °C, 400 °C, 600 °C and 800 °C were shown in Figure 13. The flexural strength of the specimen decreased with the increase in temperature. However the glass powder replacement caused a positive influence on the flexural strength at higher temperature, the strength reduction was linear for gradually cooled specimen in air. The water cooling caused a sudden reduction in the temperature leading to decrease of flexural strength than the air cooled specimen. This may be realized that the negative influence on the flexural strength is due to the reduction in the moisture of the interstitial pore water that leads to the rapid disintegration. At 200 °C the residual strength of the self-compacting glass mortar specimen showed insignificant variation in flexural performance than the unmodified self-compacting mortar. However the strength increased for a temperature of about 200 °C and 400 °C and this may be explained by the fact that the reduction in pores due to the filling of glass powders after heated.

The flexural strength decrease was almost similar to the earlier research works and proved that nanosilica showed insignificant contribution to the self-compacting glass mortar at elevated temperatures.

**Figure 14:**
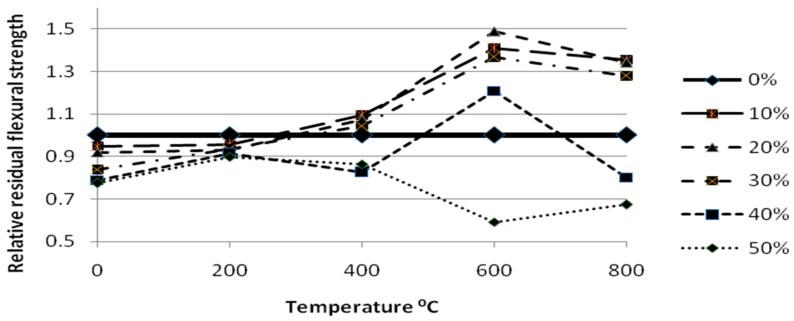
Relative residual flexural strength of gradually cooled SCGM at high temperature.

The air cooled specimens showed a little disintegration than the water cooled specimens by visual observation. It was very difficult to measure the flexural tests for the 800 °C specimens since they were completely disintegrated. The relative residual flexural strength of gradually cooled self-compacting glass mortar at high temperature was shown in Figure 14.

## 5. Conclusions

When the fresh state properties were taken into account the glass powder replaced self-compacting mortars provided acceptable values in the mini-cone and mini v funnel values. Thus the observed changes in the values obtained with comparison to the reference values were acceptable. Thus the influence of glass powder as a complete substitution of sand do not cause any detrimental effects on the fresh state characteristics which is the most important property taking into account of the practical applications.

The compressive strength result of the glass powder replaced mortars showed a fact that the glass powder has a detrimental effect on the compressive strength. Though the values decreased with increase in the glass powder proportion the values show that specimen do not suffer a significant strength loss and thus the strength achieved is more or less acceptable to a certain margin. However the nanosilica coupled glass powder replacement more or less balanced the negative influence caused by glass powder on the compressive strength of self-compacting mortar.

The water absorption and porosity values shows glass powder has a positive effect on the performance in self compacting mortar. Thus it can be concluded that the self-compacting glass mortar can be applied in areas especially subjected to humid condition.

The temperature variations also concluded the fact that the glass powder though weak in compression and normal temperature exhibited a greater positive effect when subjected to higher temperature. Thus the incorporation of glass powder can be used to enhance the thermal properties of self-compacting mortar.

The flexural strength results also showed that the flexural strengths do not increase due to glass powder replacement but the temperature increase causes a little positive influence.

The final conclusion can also be drawn that the perfect replacement in self compacting mortar for sand is glass powder when all the properties of fresh and hardened state were taken into account. The observed disadvantages in the strengths due to the addition of glass powder were almost nullified by the addition of 3% nanosilica. The air cooled specimen exhibits a greater performance than sudden cooling by water. The further studies can be done to analyze the crystalline nature of the products formed due to the glass powder substitution and temperature effects on the properties with the changes in the microstructure. However these mechanical tests performed brings in the conclusion that glass powder which is a waste product causing a great disposal problem can effectively be incorporated into the construction industry especially to enhance the self-compacting nature of concrete and an enhanced fire resistance property.

## Figures and Tables

**Table 1:** Properties of Cement.

Grade	OPC-43 Grade
Consistency	30%
Initial Setting Time	39 min
Final Setting Time	220 min
Fineness	5%

**Table 2:** Chemical Composition (mass %) of Glass Powder (GP).

SiO_2_	Al_2_O_3_	Fe_2_O_3_	MnO	CaO	Na_2_O	K_2_O	TiO_2_	P_2_O_5_	LOI
70	2	<0.1	-	6	20	-	<0.1%	-	-

**Table 3:** Mortar mix proportion.

Constituent	SCM
Cement (kg/m^3^)	700
Sand (kg/m^3^)	1372.4
Water (kg/m^3^)	276
Superplasticizer (SP) (kg/m^3^)	10.6
W/B (water/binder)	0.4

## References

[1] Ana M.M., Joana S.-C. (2012). Durability of mortar using waste glass powder as cement replacement. Constr. Build. Mat..

[2] Vijayakumar G., Vishaliny H., Govindarajulu D. (2013). Studies on Glass Powder as Partial Replacement of Cement in Concrete Production. Int. J. Emerg. Technol. Adv. Eng..

[3] Vitoldas V., Evaldas S., Harald H. (2014). The effect of glass powder on the microstructure of ultra high performance concrete. Constr. Build. Mat..

[4] Ali E.E., Al-Tersawy S.H. (2012). Recycled glass as a partial replacement for fine aggregate in self compacting concrete. Constr. Build. Mat..

[5] Sharifi Y., Houshiar M., Aghebati B. (2013). Recycled glass replacement as fine aggregate in self-compacting concrete. Front. Struct. Civ. Eng..

[6] Aliabdo A.A., Elmoaty A.E.M.A., Aboshama A.Y. (2016). Utilization of waste glass powder in the production of cement and concrete. Constr. Build. Mat..

[7] Cai Y., Xuan D., Poon C.S. (2019). Effects of nano-SiO_2_ and glass powder on mitigating alkali-silica reaction of cement glass mortars. Constr. Build. Mat..

[8] Lee H., Hanif A., Usman M., Sim J., Oh H. (2018). Performance evaluation of concrete incorporating glass powder and glass sludge wastes as supplementary cementing material. J. Clean. Prod..

[9] Patel D., Tiwari R.P., Shrivastava R., Yadav R.K. (2019). Effective utilization of waste glass powder as the substitution of cement in making paste and mortar. Constr. Build. Mat..

[10] Pan Z., Tao Z., Murphy T., Wuhrer R. (2017). High temperature performance of mortars containing fine glass powders. J. Clean. Prod..

[11] Hendi A., Mostofinejad D., Sedaghatdoost A., Zohrabi M., Naeimi N., Tavakolinia A. (2019). Mix design of the green self-consolidating concrete: Incorporating the waste glass powder. Constr. Build. Mat..

[12] Elaqra H., Rustom R. (2018). Effect of using glass powder as cement replacement on rheological and mechanical properties of cement paste. Constr. Build. Mat..

[13] Vandhiyan R., Ramkumar K., Ramya R. (2013). Experimental study on replacement of cement by glass powder. Int. J. Eng. Res. Technol..

[14] Khaliq W., Khan H.A. (2015). High temperature material properties of calcium aluminate cement concrete. Constr. Build. Mat..

[15] Culfik M.S., Ozturan T.D. (2002). Effect of elevated temperatures on the residual mechanical properties of high-performance mortar. Cement Concr. Res..

[16] Poon C.-S., Azhar S., Anson M., Wong Y.-L. (2001). Comparison of the strength and durability performance of normal- and high-strength pozzolanic concretes at elevated temperatures. Cement Concr. Res..

[17] Chan Y.N., Luo X., Sun W. (2000). Compressive strength and pore structure of high-performance concrete after exposure to high temperature up to 800 ºC. Cement Concr. Res..

[18] Le H.T., Siewert K., Ludwig H.-M. (2015). Alkali silica reaction in mortar formulated from self-compacting high performance concrete containing rice husk ash. Constr. Build. Mat..

[19] Helal M.A., HeizaKh M. (2012). Effect of fire and high temperature on the properties of the self compacted concrete. Adv. FRP Comp. Civil. Eng..

[20] Fares H., Noumowe A., Remond S. (2009). Self-consolidating concrete subjected to high temperature Mechanical and physicochemical properties. Cement Concr. Res..

[21] Zuhair M., Deshmukh S.K. (2014). Self Compacting Concrete at Elevated Temperatures—A Literature Review. Int. J. Adv. Found. Res. Sci. Eng..

[22] Vasusmitha R., Rao P.S. (2012). Effect of Elevated Temperature On Mechanical Properties Of High Strength Self Compacting Concrete. Int. J. Eng. Res. Technol..

[23] Safi B., Sadi M., Daoui A., Bellal A., Mechekak A., Toumi K. (2015). The use of sea shells as a fine aggregate (by sand substitution) in self compacting mortar (SCM). Constr. Build. Mat..

[24] Safi B., Saidi M., Aboutaleb D., Maallem M. (2015). The use of plastic waste as fine aggregate in the self compacting mortars: Effect on physical and mechanical properties. Constr. Build. Mat..

[25] Farzadnia N., Abdullah A., Ali A., Demirboga R. (2013). Characterization of high strength mortars with nano alumina at elevated temperatures. Cement Concr. Res..

[26] Bastami M., Baghbadrani M., Aslani F. (2014). Performance of nano-Silica modified high strength concrete at elevated temperatures. Constr. Build. Mat..

[27] Horszczaruk E., Sikora P., Cendrowski K., Mijowska E. (2017). The effect of elevated temperature on the properties of cement mortars containing nano-silica and heavyweight aggregates. Constr. Build. Mat..

[28] Khotbehsara M.M., Mohseni E., Yazdi M.A., Sarker P., Ranjbar M.M. (2015). Effect of nano-CuO and fly ash on the properties of self-compacting mortar. Constr. Build. Mat..

[29] Morsy M.S., Al-Salloum Y.A., Abbas H., Alsayed S.H. (2012). Behavior of blended cement mortars containing nano-metakaolin at elevated temperatures. Constr. Build. Mat..

[30] Nadeem A., Memon S.A., Lo T.Y. (2014). The performance of Fly ash and Metakaolin concrete at elevated temperatures. Constr. Build. Mat..

[31] Pathak N., Siddique R. (2012). Effects of elevated temperatures on properties of self-compacting-concrete containing fly ash and spent foundry sand. Constr. Build. Mat..

[32] Heikal M., Al-Duaij O.K., Ibrahim N.S. (2015). Microstructure of composite cements containing blast-furnace slag and silica nano-particles subjected to elevated thermally treatment temperature. Constr. Build. Mat..

[33] Mohammed A., Sanjayan J.G., Nazari A., Al-Saadi N.T.K. (2017). Effects of graphene oxide in enhancing the performance of concrete exposed to high-temperature. Austr. J. Civ. Eng..

[34] Mandandoust R., Mohseni E., Mousavi S.Y., Namnevis M. (2015). An experimental investigation on the durability of self-compacting mortar containing nano-SIO2, nano-Fe2O3 and nano-CuO. Constr. Build. Mat..

[35] Rao S., Silva P., De Brito J. (2015). Experimental study of the mechanical properties and durability of self-compacting mortars with nano materials (SiO_2_ and TiO_2_). Constr. Build. Mat..

[36] Ling T.-C., Poon C.-S., Kou S.-C. (2012). Influence of recycled glass content and curing conditions on the properties of self-compacting concrete after exposure to elevated temperatures. Cement Concr. Comp..

[37] EN Composition, Specifications and Conformity Criteria for Common Cements.

[38] BS EN Aggregates for Concrete.

[39] Schwartzentruber A., Catherine C. (2000). Method of the concrete equivalent mortar (CEM)—A new tool to design concrete containing admixture. Mater. Struct..

[40] Shi C., Wu Z., Lu K., Wu L. (2015). A review on mixture design methods for self-compacting concrete. Constr. Build. Mater..

[41] Nunes S., Matos A.M., Duarte T., Figueiras H., Joana S.-C. (2013). Mixture design of self-compacting glass mortar. Cement Concr. Comp..

[42] ASTM Standard Test Method for Flexural Strength of Hydraulic-Cement Motars.

[43] BS EN Methods of Test for Mortar for Masonry, Part 19. Determination of Water Vapour Permeability of Hardened Rendering Mortars.

